# Residual Compressive Strength of Recycled Aggregate Concretes after High Temperature Exposure

**DOI:** 10.3390/ma13081981

**Published:** 2020-04-23

**Authors:** Francisco B. Varona, Francisco Baeza-Brotons, Antonio J. Tenza-Abril, F. Javier Baeza, Luis Bañón

**Affiliations:** Department of Civil Engineering, University of Alicante, 03690 Alicante, Spain; fbaeza.brotons@ua.es (F.B.-B.); ajt.abril@ua.es (A.J.T.-A.); lbanon@ua.es (L.B.)

**Keywords:** recycled concrete, sustainability, fire engineering, high temperature, mechanical properties

## Abstract

Sustainability requirements are gaining importance in the construction industry, which needs to take specific measures in the design and construction of concrete structures. The use of recycled aggregates in concrete may be of special interest. Recycling a construction waste will close the life cycle of the original materials (e.g., concrete). Thus, environmental benefits would come from the lower waste generation, and from a lower necessity of raw materials for new structures. The current Spanish code for structural concrete considers the use of recycled aggregates in replacement rates up to 20% by aggregate mass, assimilating their properties with those of concretes without aggregate replacement. Higher substitution percentages would require further testing. In this work, substitution of coarse aggregate for recycled aggregates (with replacement percentages of 25%, 50% and 100%) has been studied, and the concrete’s residual properties after exposure to high temperatures (between 350 °C and 850 °C) have been assessed. Compressive strength and capillary water absorption tests were made after heating, and the experiments showed higher residual strength in concretes with the greatest content of recycled aggregates. However, a statistical analysis made with additional data available in the literature seemed to predict otherwise, and the recycled aggregate replacement would have a negative effect on the residual strength.

## 1. Introduction

The growing interest in the sustainable management of production processes is of particular relevance to justify special measures in the design and construction of civil infrastructure and buildings. The use of waste materials in construction is in line with the principles of sustainable development, and there exists a number of environmentally friendly production technologies that make use of waste material as fillers or additions to concrete and other construction materials, such as asphalt [1,2,3,4,5]. Among the different existing alternatives, the use of concrete with recycled aggregates, which incorporate aggregates from construction and demolition waste, is particularly relevant. This option is possible due to the existence of numerous concrete structures that are either beyond their useful life and require demolition, or have been rendered unusable by extraordinary actions, such as earthquakes. The need to process the demolition waste makes the recycled aggregates available to the construction companies. The favourable impact of this offer is twofold, because it not only gives out a product that lessens accumulation in landfills, but also makes it possible to reduce the consumption of natural aggregates, and indirectly, to control the environmental impact of the exploitation or opening of new quarries [6]. It is thus possible to ideally close the cycle of the concrete production process, with an obvious benefit from the point of view of sustainable economic development. The current Spanish Standard on Structural Concrete (EHE-08) [7] admits replacement percentages of up to 20% by mass of the coarse aggregate with recycled product, provided there are no changes to the mechanical properties and structural behaviour models established for conventional concretes. Higher replacement percentages should be supported by further experimental tests to control how the corresponding physical and mechanical properties would be affected.

The high-temperature behaviour of structural concrete has been studied for more than a century: one of the earliest scientific contributions on this topic is [8]. In the general case of conventional normal strength concretes, and even for high strength concretes, the evolution of their physical and mechanical properties under elevated temperatures has been thoroughly studied and is sufficiently defined [9,10,11,12,13,14,15,16,17,18,19]. Based on these studies, it can be concluded that the compressive strength of concrete is reduced under a high temperature, which is caused by the irreversible dehydration reactions of its components (CSH gel and portlandite) and is also negatively affected by incompatibility of thermal strains (aggregates, cement paste, reinforcements, fibres) and micro-cracking. Other properties, such as the modulus of elasticity and the tensile strength are more severely affected and are almost completely and linearly reduced in the range of 200–600 °C. Tests conducted after cooling (residual tests) arguably yield poorer mechanical properties, compared to tests obtained at high temperature [12]. Additionally, sudden cooling by quenching with cold water spraying or immersion may lead to even lower values of the residual compressive strength. Another critical issue at high temperature is the possibility of spalling, sometimes in an explosive way [11,13,14]. Spalling is associated with the pressure build-up within the concrete pores at high temperature (water and CO_2_ are released by the dehydration of the cement paste and by the calcination of limestone). Compact micro-structure concretes are more sensitive to this problem (e.g., high strength concrete, self-compacting concrete). Some of these studies [15,16,17,18,19] have tried to study whether spalling may be controlled by fibre addition or not.

In the case of concrete made with aggregates from recycled aggregate concrete (RAC), it constitutes a more recent product, and therefore, there are fewer experimental studies on its behaviour after exposure to high temperatures. Chronologically, the first study was carried out by Xiao and Zhang [20], who compared the behaviour of concretes made with natural aggregate concrete (NAC) of siliceous type with that of RAC made with aggregates coming from the demolition of an airport runway in disuse. In the exposure to temperatures up to 800 °C, no spalling was observed either in the NAC or in the RAC. The residual resistance (after cooling) of the RACs with replacement percentages of 50% was higher than that of the NACs and that of the RACs with percentages of 30%. In a subsequent study by Xiao et al. [21], the following conclusions were drawn: (i) in the range 300-500 °C, RACs exhibited a gain in compressive strength with temperature increase, as opposed to NACs; (ii) with replacements of 30%, the residual strength of RAC could be lower than that of NAC, as opposed to replacements of 50% or more; (iii) the higher the percentage of replacement, the clearer this trend was; and (iv) in the case of residual flexural-tensile strength, the percentage of replacement did not seem to have a clearly decisive influence.

Another alternative to the supply of recycled aggregates from the demolition of existing works is the direct manufacture of the recycled aggregate in the laboratory, which also ensures absolute control of its composition. This was the case in the study by Zega and Di Maio [22]. The recycled aggregates came from the demolition of test samples that had been previously manufactured in the laboratory, which enabled a study of the NAC with a specific type of aggregate (granite, siliceous and quarzitic) with the corresponding RAC with the same nature of aggregate in origin. The concretes that performed best were those manufactured with a mixture of natural quartz aggregates and recycled quartz-based aggregates. It was also observed that RACs with low water/cement ratios had better fire resistance than NACs. The study by Vieira et al. [23] also used recycled aggregates from the demolition of concrete manufactured in the laboratory, this time with limestone aggregates. Unlike the previous references, in this case no conclusive results were observed on a possible correlation between replacement percentage and better or worse behaviour at high temperatures.

The question of whether RACs perform better at high temperatures than NACs is not entirely clear. Previous and more recent studies by Sarhat and Sherwood [24] would seem to support that RACs display better compressive strengths after exposure to high temperatures, even compared to NACs of limestone aggregates above 500 °C. This work also seemed to support the hypothesis by [20,21]: that replacements above 50% would yield even better results. However, there are experimental investigations that would undermine these conclusions, as in the case of Gupta et al. [25], who compared laboratory-made RAC with a NAC with basaltic aggregates. One of the reasons that could justify the hypothesis of the RACs having the best behavior in the event of a fire is that they have lower thermal conductivity, and therefore, a lower penetration rate of the temperature towards the interior of the cross section. This fact could be observed in experimental tests on full-scale reinforced concrete columns carried out by Dong et al. [26]. Table 1 summarizes the main experimental campaigns carried out on RAC at high temperatures, breaking down, by columns, the most relevant variables under study.

It can be concluded that most studies focused on conventional strength concretes, with a control NAC made with siliceous aggregates, tested at an early age and exposed to high temperatures in an electric furnace, with heating rates lower than those associated with direct exposure to fire. The usual maximum temperature is 800 °C, yet the duration of the plateau of thermal saturation at the maximum temperature is very variable (from one to six hours) and is not related to the sizes of the test specimens. This aspect of the experimental methodology can pose questionable situations, such as the case of [22], in which, with a maximum exposure temperature of 500 °C maintained for one hour, the thermocouples inside the test samples did not exceed 265 °C. Finally, the vast majority of the experimental campaigns included in Table 1 determined the residual values of the mechanical properties of the concrete; i.e., once cooled down to room temperature after having been subjected to the target temperature of each step. Regarding the compressive strength tests, many studies did not report the respective standards according to which the tests were conducted. Nonetheless, the EN 12390-3 standard was used in [23,31], the ASTM C 39 was used in [22,24,27,39,41], the GB/T 50081-2002 was used in [32,40] and the BS 1881-116 was used in [28].

Some of the earlier studies on the compressive strength of RAC under fire found it to be superior to that of NAC [20,21,22,42,43], especially with replacement percentages equal or greater than 50%. A recent study on high strength concrete with recycled aggregates [38] also reported its better performance than that of high strength concrete with natural aggregates, in terms of both relative mechanical properties and physical stability at elevated temperatures. Other studies have not found the replacement percentage to have a significant impact [23,36,39,44]. Furthermore, in some recent studies [35,40,41] RACs exhibited greater reductions in compressive strength at high temperatures than their counterpart control concretes containing natural aggregates.

Given the uncertainty around the exact effects that replacement of a natural aggregate with a recycled product has on the performance after exposure to elevated temperatures, the purpose of this work was to study, experimentally, the behaviours of concretes manufactured with recycled aggregates, with different replacement percentages, cooled after exposure to high temperatures. This information was gathered to answer the question of whether or not concretes with recycled aggregates offer a better residual behaviour after exposure to high temperatures.

## 2. Materials and Methods

The recycled aggregates used in this study came from the Arecosur plant (Málaga, Spain), dedicated to the treatment of construction and demolition waste (CDW). The natural aggregates used in NAC concrete and in combination with RAC were limestone in nature, from the Santa Rita VI quarry in Abanilla-Murcia (Spain). The particle size of the natural aggregates was 6/12 mm (determined in accordance with EN 933-1). The recycled aggregates supplied by Arecosur had a wide range of sizes, so they had to be sieved to make them compatible with the size of natural aggregates. Four different batches were prepared: one for the control, with 100% of the coarse aggregate composed of natural limestone (A0); and three mixes with different replacement percentages of the coarse aggregate with recycled product (A25 with 25%, A50 with 50% and A100 with 100%). Details of the composition of each batch are shown in Table 2. Twenty-four cubic specimens of 10 cm in size were produced from each of the mixes and remained in a wet chamber under controlled environmental conditions for up to 28 days (20 ± 1 °C and relative humidity equal to or greater than 95%). The cement type specified in Table 2 is in accordance with EN 197-1.

The fibres added to the concrete mix were made of natural polypropylene (PP) monofilament, 12 mm long and with a diameter between 31 and 35 microns. Their use was aimed at controlling explosive spalling during the heating stage. The melting temperature of polypropylene is between 163 and 170 °C. These values are well below the temperature ranges in which the dehydration reactions of CSH gel and portlandite occur, which contribute to the increase in pore pressure. The capillary net that appears once the polypropylene melts serves as a reliever for the pore pressure and prevents explosive spalling [16,17,18,19].

**Figure 1:**
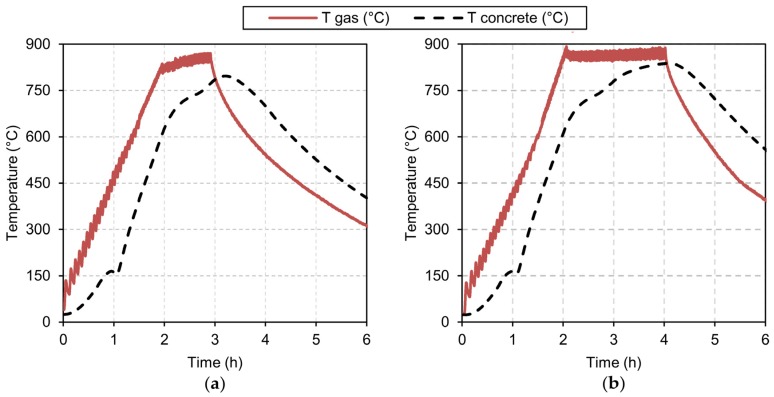
Time evolution of temperature during heating process: (**a**) thermal saturation during 1 hour; (**b**) thermal saturation during 2 hours.

After the specimens were cured in a wet chamber for 28 days, they were kept until the time of testing in a laboratory environment (20 ± 2 °C and relative humidity between 55% and 65%). The tests at room temperature and high temperature exposure were carried out when the specimens had an age of 110 days for all batches. The heating process was carried out in an electric furnace with a volume of 1 m^3^, capable of reaching and maintaining temperatures of 1200 °C. The following target temperature values were selected: 350, 550, 700, 775 and 850 °C. The heating rate was adjusted to 7–7.5 °C/min. Once the target temperature was reached in each program, it had to be maintained for a period of time in order to thermally stabilize the interior of the concrete specimens. At the end of this thermal saturation plateau, the furnace switched off its resistors and the specimens were kept inside and naturally cooled. Given the great diversity in the durations of the aforementioned saturation plateaus in the experimental campaigns summarised in Table 1, it was decided to carry out a preliminary study to try to determine the required duration. For this purpose, additional specimens of the A0 control mix were manufactured; thermocouples were placed at the centre of these specimens to test the evolution of the temperature during the heating process. An additional thermocouple checked the temperature of the air inside the electric furnace. These readings were made on 118 day old specimens, in the range of the specimens that were going to be subjected to the high temperature exposure tests. The thermocouple readings are shown in Figure 1. It can be seen that, despite the small size of the specimen (10 cm), its centre barely reached 800 °C with a maximum outside temperature of 850 °C maintained for one hour. But if the saturation plateau was extended to two hours, then the centre of the specimen reached 838 °C, which can be considered a value sufficiently close to that of the target temperature. In addition, the centre of the specimen exceeded 95% of the maximum target temperature for 73 minutes. For these reasons, in the final heating tests, it was decided to set two hours as the duration of the saturation plateau for all specimens.

After cooling of the specimens, the residual properties characterization tests were carried out in parallel to the tests conducted on specimens that had not been exposed to high temperatures. These were the following: measurement of the dry density of the specimens (*ρ_c_*); capillary water absorption tests; compressive strength tests; and scanning electron microscopy (SEM) photographs.

Compressive strength tests were carried out in accordance with standard EN 12390-3. The test speed was adjusted to 0.3 t/s (equivalent to 0.3 MPa/s in the cross section of the specimens). Three specimens were tested at each target temperature (20, 350, 550, 700, 775 and 850 °C). Capillary water absorption tests were done in accordance with Spanish standard UNE 83982. This test involves determining the rate of water absorption by recording the increase in mass of specimens due to the absorption of water as a function of time when only one side of the specimen is exposed to water. The exposed surface is submerged in a 5 ± 1 mm sheet of water. The masses of the specimens were recorded at 5, 10, 15, 30, 60, 120, 180, 240 and 360 minutes and 24 hours after the initial contact with water, thereby giving us both the gain in mass as a function of the time and the capillary absorption coefficient. The capillary water absorption results were based on one specimen for each target temperature, differently from those used for the compressive strength experiments. Therefore, the total number of specimens in this study was 96 (24 samples for each batch A0, A25, A50 and A100).

Finally, the morphologies of the concretes subjected to different temperatures were also examined with a scanning electron microscope (SEM). For the study of the samples, small fragments of all the concretes were extracted after subjecting them to the target temperature. To observe acceptable SEM images and to preserve the structures of the samples placed directly in high vacuum, they were dried beforehand in an oven at 60 °C for 24 hours to remove any moisture (a wet sample exposed to vacuum might lose its water in an uncontrolled dehydration process, which often distorts or destroys the structure of the sample). Subsequently, the samples were metallized with Au-Pd (30 nm) in order to improve the image quality. The images were taken at high vacuum, under 15 kV and from a variable working distance.

## 3. Results and Discussion

**Figure 2:**
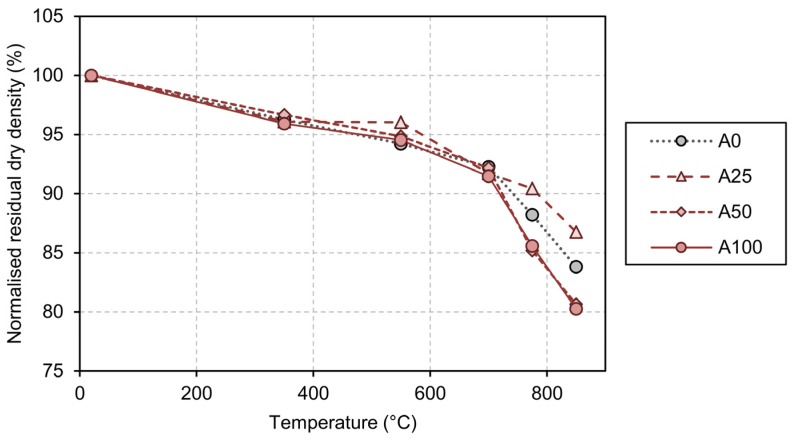
Evolution of density of natural aggregate concrete (NAC) and RAC with temperature.

The results of the tests related to density variation are presented first in Table 3 as a function of temperature and replacement percentage, as well as graphically in Figure 2. Table 3 also includes the 90% bi-lateral confidence interval (95% uni-lateral), based on 24 measurements (at 20 °C) or four measurements (for temperatures equal or higher than 350 °C). In the case of dry density *ρ_c_*, it can be seen that it is not significantly affected up to temperatures of 700 °C, and there are no noticeable differences in behaviour between the NAC and the different RACs. At low exposure temperatures (not greater than 300 °C) the mass loss can be attributed to the evaporation of the water content within the concrete, though not combined with the cement paste components (portlandite and CSH gel). That is the reason why all concrete batches show an almost identical evolution. At very high temperatures, especially in excess of 700 °C, there is a visible deterioration in density, which in this case is greater in the RACs with high replacement percentages (A50 and A100). The mass loss associated with temperatures in excess of 550 °C can be attributed to the water content that is lost in the dehydration reactions that affect the CSH gel and the portlandite of the cement paste. The reason why A50 and A100 exhibit greater mass loss is that a considerable part of their recycled coarse aggregate contains old cement paste, which suffers the same chemical dehydration reactions that affect the newer cement paste, thereby contributing to a greater mass loss than A0, which only contains a newer cement paste.

**Figure 3:**
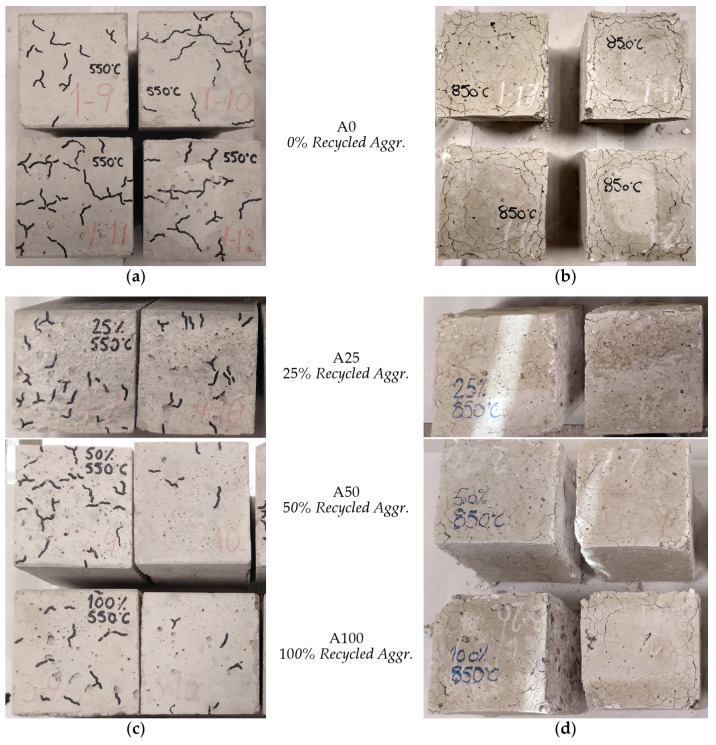
External cracking of specimens after high temperature exposure: NAC batches at (**a**) 550 °C and at (**b**) 850 °C; RAC batches at (**c**) 550 °C and at (**d**) 850 °C.

Before the destructive tests were carried out, a qualitative comparative study was also carried out through a naked-eye analysis of the external cracking of the specimen surfaces caused by exposure to high temperatures. This can be seen in the photographs shown in Figure 3a,b, corresponding to NAC concrete (A0) at temperatures of 550 and 850 °C, respectively, and in Figure 3c,d, corresponding to RAC concretes at the same exposure temperatures. In the range of 550 °C, it can be seen that the density of cracks is reduced in the RAC with replacements of 50% and 100%. When specimens were exposed to 850 °C, the degree of cracking was very pronounced, especially at edges and corners, as a result of the local effects of the incompatibility of thermal deformations. However, in the three RAC concretes the densities of cracks were slightly lower than that of NAC, which confirms the trend observed at 550 °C. The lower cracking observed in the RACs may be related to the fact that the natural aggregate has different thermal properties (expansion coefficient, conductivity and specific heat) to those of the cementitious matrix in which it is incorporated. Consequently, its replacement by recycled aggregates, which incorporate an older, adhered cementitious matrix, reduces thermal incompatibilities.

The results of the compressive strength tests (*f_c,cub_*) are shown in Table 4 as a function of the target exposure temperature and of the recycled aggregate replacement percentage. The statistical dispersion is addressed by also including the 90% bi-lateral (95% uni-lateral) confidence intervals in the results annotated in Table 4. At ambient temperature it can be observed that the RACs tend to reduce the compressive strength with respect to the NAC (A0). This may be due to the manufacturing process of the concretes of this campaign, in which the recycled aggregates were not saturated with water prior to their mixing with the rest of the components of the concrete. Therefore, their porosity must have contributed to reducing the amount of hydration in the cement with respect to the A0 control mix. Nevertheless, one result that is difficult to explain is that the impact of the percentage of replacement on the compressive strength does not seem to follow a proportional law at ambient temperature: in the case of the mix with 25% recycled aggregate (A25) a 26% reduction in strength was obtained, while for higher replacements (A50 and A100) it was only 10%. In contrast, the effect of the percentage replacement of coarse aggregate with recycled aggregate appears to be beneficial from the point of view of residual compressive strength at high temperatures. In the 350 °C range the NAC (A0) loses 22% resistance, while the RAC loses only between 2.5% and 12% (the smaller loss corresponds to 100% replacement with recycling). At higher temperatures, in the 550 °C range, the trend seems to continue, with NAC losing almost 50% of resistance, while RACs would retain between 56% and 72%, and again, the best performance is that of A100. At higher temperatures the trend remains similar, with the highest residual strength measured at A100 (even twice that of NAC at or above 775 °C).

**Figure 4:**
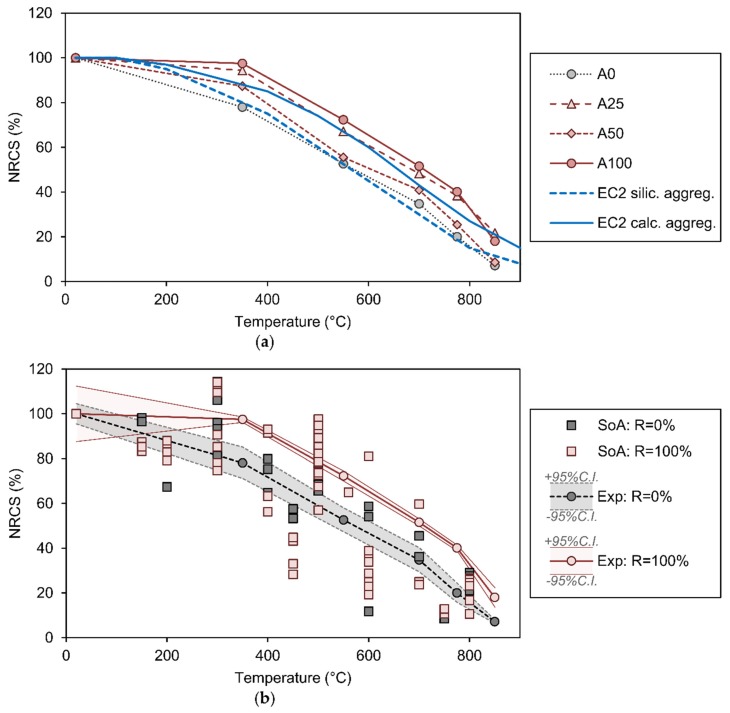
Evolution of normalised compressive strength (NRCS) with high temperatures: (**a**) experimental results for replacements of 0%, 25%, 50% and 100%, compared with NAC curves from Eurocode 2 [21]; (**b**) experimental results for replacements of 0% and 100%, compared with results from state-of-the-art (SoA).

The evolution of *f_c,cub_* with temperature is represented graphically in Figure 4a. The vertical axis of this graph represents the normalized residual compressive strength (*NRCS*) which is the quotient of the residual resistance after exposure to high temperature (measured on the horizontal axis) with respect to the original ambient temperature resistance of the corresponding batch. This graph not only represents the experimental results obtained in this campaign, but also the evolution curves of compressive strength at temperature proposed in Eurocode 2, Part 1–2 [45]. As can be seen, there are two different curves: one for conventional resistance concretes with siliceous aggregates and another for those manufactured with limestone aggregates. The former have worse fire behaviour due to their higher thermal conductivity and the crystalline transformation from α-quartz to β-quartz that takes place around 550 °C. This transformation entails a significant volumetric increase which leads to micro-cracking of the concrete and a loss in compressive strength. Furthermore, the experimental results obtained for NAC and RAC (with replacement of 100%) are again represented in Figure 4b, but this time they are contrasted with experimental results previously reported by Zega and Di Maio [22], Xiao et al. ([21], Kou et al. [28], Gales et al. [29], Laneyrie et al. [31], Zhao et al. [36] and Chen et al. [46], which were the only experimental campaigns which did not test the specimens immediately after curing.

A first observation regarding the high temperature exposure methodology applied in this study can be extracted from the evolution of the graphs shown in Figure 4a. Despite having manufactured the NAC with limestone aggregates, its experimental behaviour at high temperature is somewhat worse than that predicted by Eurocode 2, since it seems to be close to that of a NAC with siliceous aggregates. This would demonstrate that the heating rate and thermal saturation plateau that have been adopted produce a sufficient level of deterioration in the strength of the concrete and lead to conservative results. A second observation that is evident in Figure 4a is the better performance of RACs with respect to NAC. In fact, RACs with replacement percentages of 50% and 100% would perform better than Eurocode 2 limestone NACs between 350 and 550 °C and between 700 and 775 °C.

Regarding the information graphically represented in Figure 4b, the experimental results of this research (Table 4) seem to agree with a trend which may arguably be observed in the results reported in the state-of-the-art. The best results for *NRCS* in the range 400–700 °C correspond to RAC with a replacement of 100%, whilst the worst results for *NRCS* in the range 600-800 °C correspond to NAC. The seven references that are represented in Figure 3b plus the new results of Table 4 have been used to create a database for developing a multi-variable prediction model. The independent variables that have been identified are the following:

- Maximum exposure temperature, *T*, in °C.
- Replacement percentage of coarse aggregate with recycled product, *R*.
- Age of samples at testing, in days, *A*. This variable ranges from 42 to 300 days.
- Compressive strength at 20 °C, in MPa, *f_c,_*_20_. This variable ranges from 20.8 to 97.4 MPa.
- Thermal damage parameter, *δ*, in hours/dm^2^. This variable is the ratio of duration of thermal saturation plateau to minimum size of specimen squared. This variable ranges from 0.44 to 4.

This methodology has been previously applied to develop predictive models for mechanical properties of concrete at high temperature [47]. The number of experiments available is 240 (including the experimental results in this research). Therefore, there are 48 experimental datums per independent variable. A non-linear multiple regression analysis was carried out using SPSS and a prediction model was developed, which is represented in Equation (1):

(1) $$NRCS=\left(124-\frac{1.01}{10^{2}}T-\frac{1.42}{10^{4}}T^{2}\right)\left(1.26-\frac{2.74}{10^{3}}f_{c,20}\right)\left(0.938-\frac{2.73}{10^{4}}R\right)\left(0.921-\frac{4.82}{10^{4}}A\right)\left(0.831-\frac{2.07}{10^{2}}\delta \right)$$

**Figure 5:**
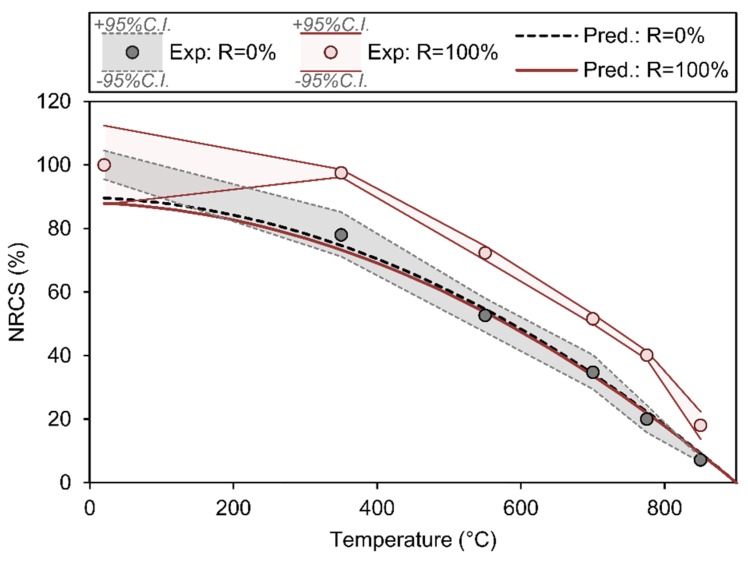
Experimental observations of concrete batches A0 (NAC, with *R* = 0%) and A100 (RAC, with *R* = 100%) and prediction models for *R* = 0% and *R* = 100%, based on a non-linear multiple variable regression analysis sourced from experiments reported here and in the state-of-the-art.

The R^2^ coefficient for this model is 0.814. One of the most striking features of this model is that the coefficient multiplying the replacement percentage *R* is negative, thereby indicating that this variable seems to have an unfavourable effect on the compressive behaviour at high temperature. Another interesting feature is that the coefficients multiplying both replacement percentage *R* and age *A* are very small, thereby suggesting that these variables may not have a statistically significant impact on *NRCS*. The experimental results presented in this paper (for *R* = 0% and *R* = 100%) and their corresponding prediction models (with *A* = 110 days, *δ* = 2 and *f_cd,_*_20_ = 46.5 MPa for *R* = 0% and *f_cd,_*_20_ = 42.0 MPa for *R* = 100%) are represented in Figure 5. Although the experimental results in Table 4 suggest that replacement with recycled aggregate is beneficial for the compressive strength after high temperature exposure, it can be clearly observed that the replacement percentage may in fact have a negligible effect according to a statistical model sourced from reported experiments.

**Figure 6:**
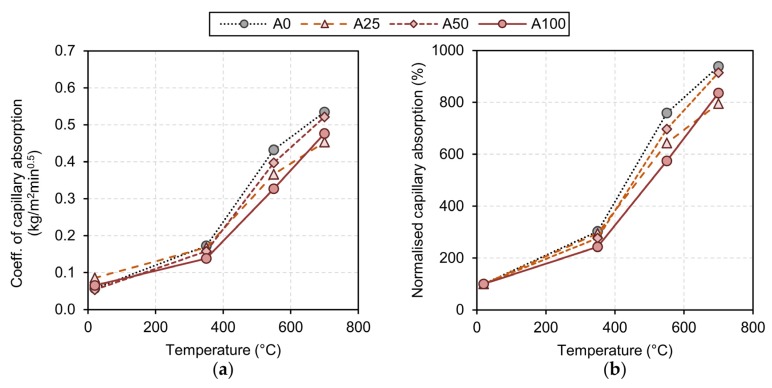
Evolution of the capillary water absorption with temperature. (**a**) Coeff. of capillary absorption (**b**) Normalised capillary absorption.

As far as the results of the capillary water absorption tests are concerned, they could be carried out up to a temperature of 700 °C. The specimens that had reached 775 °C would have crumbled directly on contact with water, making it impossible to perform these tests. The results are represented graphically in Figure 6. Up to a temperature of 350 °C there does not appear to be any significant differences in the capillary absorption coefficient of the specimens. However, at 550 °C RAC specimens appear to exhibit lower absorption (around 25% less in the case of a 100% replacement rate). At a higher temperature (700 °C), the trend is similar but less pronounced (a reduction of 11% with a 100% replacement). The evolution of the coefficient of absorption at high temperatures, which has been measured experimentally, is consistent with that reported in other studies, such as [28], wherein, after exposure to high temperatures, comparatively lower absorptions were also measured in the RAC test specimens. The reason for this behaviour is attributed to the fact that the recycled aggregate shows better thermal compatibility with the cementitious matrix; hence, the micro-cracking that occurs due to the expansion of the concrete components is attenuated.

**Figure 7:**
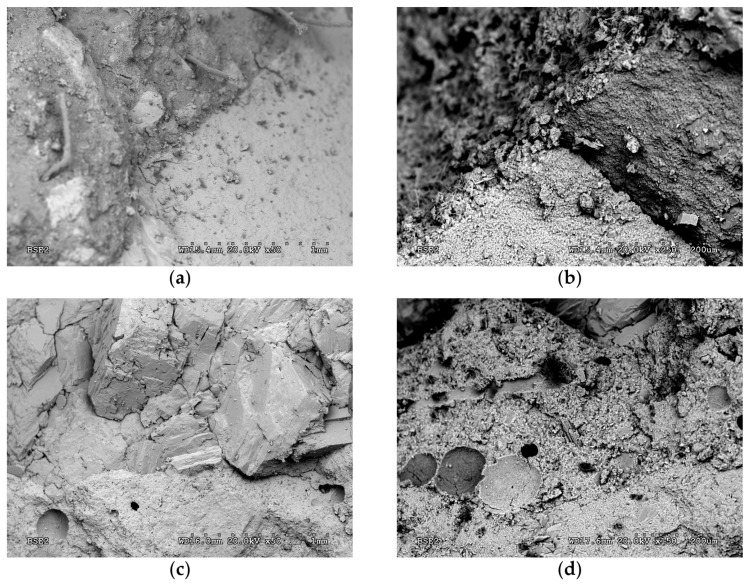
Scanning electron microscope photographs: A0 batch at room temperature (**a**) and at 550 °C (**b**); A100 batch at room temperature (**c**) and at 550 °C (**d**).

Figure 7 shows different SEM photographs of the concrete samples A0 (0% replacement) and A100 (100% replacement), both at 20 °C and 550 °C. Figure 7a shows the interface between the natural aggregate and the new cement matrix, and the polypropylene fibres. It can be seen that the interface is in perfect condition. On the contrary, after exposure to 550 °C (Figure 7b), a marked degradation of the interface between paste and aggregate is observed, with a noticeable separation.

In the case of the RAC with 100% replacement, Figure 7c shows the microstructure and its different components: the new cementitious paste, the old (recycled) paste and natural aggregates present in the recycled product. After exposure to 550 °C (Figure 7d), degradation of both new and old mortar pastes can be observed, but not a clear separation between the two of them. The new paste is clearly identified by the holes left after the melting of the polypropylene fibres. The degradation of the interface between cement pastes and aggregates present in the recycling is very perceptible in Figure 7d. This finding agrees with observations previously reported in [28] and [37], which may support the idea that NAC is prone to suffer more thermal deterioration than RAC due to wider internal mesocracks propagating around the natural aggregates.

## 4. Conclusions

The growing concern for increased sustainability in the construction industry and public works makes concrete with recycled aggregates a product of considerable attractiveness. As a consequence, it is necessity to empirical support its performance in different situations, such as during fires. As reported in this study, a review of the state-of-the-art concerning previous experimental campaigns was carried out and a particular empirical study was carried out. The main conclusions are as follows:

- The evolution of the dry density at high temperatures, of concretes with natural aggregates (NAC) and with recycled aggregates (RAC), is very consistent and does not seem to be decisively affected by the percentage of replacement with recycled aggregates up to 700 °C. More severe thermal exposures produce a remarkable loss of density.
- The experimental results presented in this paper may lead one to conclude that the replacement of coarse aggregates with recycled product has a beneficial effect on the compressive strength after high temperature exposure. This conclusion agrees with some of the previously reported research in this topic.
- A database of experimental results has been created, including the experimental results reported here as well as those described by other authors. This database has been used to develop a non-linear multivariable model, which explains the evolution of the compressive strength as a function of the exposure to high temperature, the recycled aggregate replacement percentage, the age at testing, the compressive strength at ambient temperature and a damage parameter which takes into account the duration of the exposure to high temperature and the sizes of the specimens.
- The prediction model created suggests that the replacement percentage has a negligible impact on the compressive strength after exposure to elevated temperatures.
- Capillary water absorption in both NACs and RACs increases considerably with exposure to high temperatures, being eight times greater at 700 °C. It was observed that the increase of the absorption coefficient in the RAC was slightly lower than in the NAC, which seems to be clearly attributable to the greater compatibility of the thermal properties of the new and old mortar pastes in the RAC.
- SEM photographs have shown lower levels of micro-cracking and degradation of the interfaces between new and old mortar pastes from recycled aggregates, which would support the above conclusion on greater thermal compatibility in RACs.
- The latter phenomenon also appears to be the key to justifying that residual compressive strength after fire exposure is better in RACs than in NACs. With the highest replacement percentages, the relationship between compressive strength and temperature is even above the standard prescribed for concretes with natural limestone aggregates, which are the conventional concretes expected to exhibit the best performance.

## Figures and Tables

**Table 1:** Summary of the state-of-the-art concerning experiments on recycled aggregate concrete (RAC) at high temperatures (beginning).

Reference	Grade	Natural Aggreg.	Replace-Ment	Specimen	Age	Heating	Max. T	Tests
[20]	NSC	S	30% 50% 70% 100%	Cub.	28 d	E	800 °C	C
				15 cm			2 h	
[22]	NSC	S	75%	Cyl.	140 d	E	500 °C	C, E
				15 × 30 cm			1 h	
[27]	NSC	C	100%	Cyl.	30 d	E	750 °C	C, ST, E
		S		10 × 20 cm			1 h	
[23]	HSC	C	20% 50% 100%	Cub. 15 cm Cyl. 15 × 30 cm	50 d	F	800 °C 1 h	C, ST, E
[25]	NSC	B	50%	Cub. 15 cm Cyl. 15 × 30 cm	28 d	E	1000 °C	C, σ-ε, M
			100%				6 h	
[24]	NSC	S	25% 50% 75% 100%	Cyl.	30 d	E	750 °C	C, ST, E
		C		10 × 20 cm			1 h	
[21]	NSC	S	30%	Cub.	42 d	E	800 °C 2 h	C, FT
			50%	15 cm				
			70%	Prism.				
			100%	10 × 10 × 40				
[26]	NSC	-	100%	Column	-	F	1000 °C	C, T, D
				RC			4 h	
[28]	NSC	S	50%	Cub. 10 cm Cyl. 10 × 20 cm	90 d	E	800 °C	C, ST, WA
			100%				4 h	
[29]	NSC	C	30%	Cub.	180 d	E	500 °C	C, E, M
			100%	10 cm			2 h	
[30]	NSC	S	15%	Cub.	7 d	E	600 °C	C, D
			30%	15 cm	28 d		2 h	
[31]	NSC	S/C	100%	Cyl.	90 d	E	750 °C	C, ST, E, D, T
	HSC			11 × 22 cm			2 h	
[32]	NSC	C	30%	Prism.	28 d	E	800 °C	C, σ-ε
		NC	50%	15 × 15 × 30			4 h	
[33]	NSC	C	30% 50% 70% 100%	Cub.	28 d	E	500 °C	C, σ-ε
				15 cm			6 h	
[34]	NSC	-	30% 70% 100%	Cyl.	28 d	E	500 °C	TC
	HSC			10 × 20 cm			1 h	
[35]	NSC	S	50%	Cyl.	30 d	E	800 °C	C, E
			100%	10 × 20 cm			1 h	
[36]	NSC	S	25% 50% 75% 100%	Prism.	300 d	E	800 °C	C, σ-ε
				10 × 10 × 30			3 h	
[37]	NSC	S	20% 40% 60% 80% 100%	Cyl.	30 d	E	800 °C	C, σ-ε, Th, M
				10 × 20 cm			2 h	
[38]	HSC	C	100%	Cyl.	28 d	E	800 °C	C, ST, σ-ε, D, Th, M
				10 × 20 cm			2.5 h	
[39]	HSC	C	15%	Cyl.	150 d	E	600 °C	C
			30%	11 × 22 cm			2 h	
[40]	NSC	-	30% 50% 70% 100%	Prism.	60 d	E	800 °C	C, D, σ-ε
				15 × 15 × 30			1 h	
[41]	NSC	-	30% 60% 100%	Cyl.	28 d	E	600 °C	C, ST, D
				10 × 20 cm			1 h	

Grade: normal strength concrete, *NSC*; high strength concrete, *HSC*. Natural aggregate: siliceous, *S*; calcareous, *C*; basalt, *B*; non-conventional, *NC*. Heating: electric furnace, *E*; fire exposure, *F*. Tests: compressive strength, *C*; splitting tensile strength, *ST*; flexural-tensile strength, *FT*; triaxial compressive strength, *TC*; elastic modulus, *E*; stress-strain relationship, *σ-ε*; density, *D*; electron microscopy, *M*; capillary water absorption, *WA*; Th, thermal properties.

**Table 2:** Compositions of the tested concrete batches.

Component	A0	A25	A50	A100
CEM II/A-V 42.5R (kg/m^3^)	270	270	270	270
W/C	0.65	0.65	0.65	0.65
Natural sand (kg/m^3^)	750	750	750	750
Natural aggregate 6/12 mm (kg/m^3^)	1156	289	578	-
Recycled aggregate 6/12 mm (kg/m^3^)	-	867	578	1156
Natural polypropylene fibres (kg/m^3^)	2	2	2	2

**Table 3:** Evolution of dry density at high temperatures.

T (°C)	Dry Density (kg/m^3^)			
	A0	A25	A50	A100
20	2365 ± 6	2312 ± 10	2319 ± 15	2318 ± 10
350	2276 ± 18	2222 ± 20	2241 ± 45	2223 ± 16
550	2228 ± 25	2221 ± 22	2199 ± 53	2191 ± 41
700	2182 ± 17	2121 ± 26	2137 ± 20	2120 ± 30
775	2086 ± 35	2091 ± 34	1975 ± 48	1983 ± 30
850	1982 ± 9	2006 ± 36	1870 ± 40	1860 ± 61

**Table 4:** Evolution of the compressive strength at high temperatures.

Batch	Compressive Strength *f_c,cub_* (MPa)					
	20 °C	350 °C	550 °C	700 °C	775 °C	850 °C
A0	46.5 ± 2.1	36.3 ± 3.3	24.5 ± 2.5	16.2 ± 2.5	9.3 ± 1.9	3.3 ± 0.4
A25	34.4 ± 1.6	32.4 ± 1.4	23.1 ± 0.2	16.6 ± 0.7	13.2 ± 1.9	7.5 ± 1.0
A50	42.0 ± 3.2	36.6 ± 2.2	23.3 ± 3.6	17.2 ± 1.1	10.7 ± 1.2	3.6 ± 0.6
A100	42.0 ± 5.2	40.9 ± 0.5	30.4 ± 1.0	21.6 ± 0.7	16.8 ± 0.6	7.6 ± 1.8

## References

[1] Tugrul Tunc E. (2019). Recycling of marble waste: A review based on strength of concrete containing marble waste. J. Environ. Manag..

[2] Woszuk A., Wróbel M., Franus W. (2019). Influence of Waste Engine Oil Addition on the Properties of Zeolite-Foamed Asphalt. Materials.

[3] Woszuk A., Wróbel M., Franus W. (2020). Application of Zeolite Tuffs as Mineral Filler in Warm Mix Asphalt. Materials.

[4] Baeza F.J., Galao O., Vegas I.J., Cano M., Garcés P. (2018). Influence of recycled slag aggregates on the conductivity and strain sensing capacity of carbon fiber reinforced cement mortars. Constr. Build. Mater..

[5] Acosta Álvarez D., Alonso Aenlle A., Tenza-Abril A.J., Ivorra S. (2019). Influence of partial coarse fraction substitution of natural aggregate by recycled concrete aggregate in hot asphalt mixtures. Sustainability.

[6] Oikonomou N.D. (2005). Recycled concrete aggregates. Cem. Concr. Compos..

[7] Ministerio de Fomento (2011). Gobierno de España Instrucción de Hormigón Estructural (EHE-08).

[8] Lea F.C. (1920). The effect of temperature on some of the properties of materials. Engineering.

[9] Thelandersson S. (1972). Effect of high temperatures on tensile strength of concrete. Nord. Betong.

[10] Anderberg Y., Thelandersson S. (1976). Stress and deformation characteristics of concrete at high temperatures-2. Experimental Investigation and Material Behaviour Model.

[11] Castillo C., Durrani A.J. (1987). Effect of transient high temperature on high strength concrete. ACI Mater. J..

[12] Bazant Z.P., Kaplan M.F. (1996). Concrete at High Temperatures: Material Properties and Mathematical Models.

[13] Poon C.S., Azhar S., Anson M., Wong Y.L. (2001). Comparison of the strength and durability performance of normal- and high-strength pozzolanic concretes at elevated temperatures. Cem. Concr. Res..

[14] Kodur V.K.R. (2005). Guidelines for fire resistance design of high-strength concrete columns. J. Fire Prot. Eng..

[15] Lau A., Anson M. (2006). Effect of high temperatures on high performance steel fibre reinforced concrete. Cem. Concr. Res..

[16] Varona F.B., Baeza F.J., Ivorra S., Bru D. (2015). Experimental analysis of the loss of bond between rebars and concrete exposed to high temperatures. Dyna.

[17] Varona F.B., Baeza F.J., Bru D., Ivorra S. (2018). Influence of high temperature on the mechanical properties of hybrid fibre reinforced normal and high strength concrete. Constr. Build. Mater..

[18] Ding Y., Azevedo C., Aguiar J.B., Jalali S. (2012). Study on residual behaviour and flexural toughness of fibre cocktail reinforced self compacting high performance concrete after exposure to high temperature. Constr. Build. Mater..

[19] Peng G.F., Bian S.H., Guo Z.Q., Zhao J., Peng X.L., Jiang Y.C. (2008). Effect of thermal shock due to rapid cooling on residual mechanical properties of fiber concrete exposed to high temperatures. Constr. Build. Mater..

[20] Xiao J., Zhang C. (2007). Fire damage and residual strengths of recycled aggregate concrete. Key Eng. Mater..

[21] Xiao J., Fan Y., Tawana M.M. (2013). Residual compressive and flexural strength of a recycled aggregate concrete following elevated temperatures. Struct. Concr..

[22] Zega C.J., Di Maio A.A. (2009). Recycled concrete made with different natural coarse aggregates exposed to high temperature. Constr. Build. Mater..

[23] Vieira J.P.B., Correia J.R., De Brito J. (2011). Post-fire residual mechanical properties of concrete made with recycled concrete coarse aggregates. Cem. Concr. Res..

[24] Sarhat S.R., Sherwood E.G. (2013). Residual mechanical response of recycled aggregate concrete after exposure to elevated temperatures. J. Mater. Civ. Eng..

[25] Gupta A., Mandal S., Ghosh S. (2012). Recycled aggregate concrete exposed to elevated temperature. Mater. Sci..

[26] Dong H., Cao W., Bian J., Zhang J. (2014). The fire resistance performance of recycled aggregate concrete columns with different concrete compressive strengths. Materials.

[27] Sarhat S.R., Sherwood E.G. The behaviour of recycled aggregate concrete at elevated temperatures. Proceedings of the Proceedings, Annual Conference-Canadian Society for Civil Engineering.

[28] Kou S.C., Poon C.S., Etxeberria M. (2014). Residue strength, water absorption and pore size distributions of recycled aggregate concrete after exposure to elevated temperatures. Cem. Concr. Compos..

[29] Gales J., Parker T., Cree D., Green M. (2016). Fire performance of sustainable recycled concrete aggregates: Mechanical properties at elevated temperatures and current research needs. Fire Technol..

[30] Salau M.A., Oseafiana O.J., Oyegoke T.O. (2015). Effects of elevated temperature on concrete with Recycled Coarse Aggregates. Proceedings of the IOP Conference Series: Materials Science and Engineering.

[31] Laneyrie C., Beaucour A.L., Green M.F., Hebert R.L., Ledesert B., Noumowe A. (2016). Influence of recycled coarse aggregates on normal and high performance concrete subjected to elevated temperatures. Constr. Build. Mater..

[32] Liu Y., Wang W., Chen Y.F., Ji H. (2016). Residual stress-strain relationship for thermal insulation concrete with recycled aggregate after high temperature exposure. Constr. Build. Mater..

[33] Yang H., Lv L., Deng Z., Lan W. (2017). Residual compressive stress-strain relation of recycled aggregate concrete after exposure to high temperatures. Struct. Concr..

[34] Meng E., Yu Y., Yuan J., Qiao K., Su Y. (2017). Triaxial compressive strength experiment study of recycled aggregate concrete after high temperatures. Constr. Build. Mater..

[35] Shaikh F.U.A. (2018). Mechanical properties of concrete containing recycled coarse aggregate at and after exposure to elevated temperatures. Struct. Concr..

[36] Zhao H., Wang Y., Liu F. (2017). Stress-strain relationship of coarse RCA concrete exposed to elevated temperatures. Mag. Concr. Res..

[37] Xuan D., Zhan B., Poon C.S. (2018). Thermal and residual mechanical profile of recycled aggregate concrete prepared with carbonated concrete aggregates after exposure to elevated temperatures. Fire Mater..

[38] Khaliq W. (2018). Taimur Mechanical and physical response of recycled aggregates high-strength concrete at elevated temperatures. Fire Saf. J..

[39] Pliya P., Cree D., Hajiloo H., Beaucour A.L., Green M.F., Noumowé A. (2019). High-Strength Concrete Containing Recycled Coarse Aggregate Subjected to Elevated Temperatures. Fire Technol..

[40] Chen Z., Zhou J., Ye P., Liang Y. (2019). Analysis on mechanical properties of recycled aggregate concrete members after exposure to high temperatures. Appl. Sci..

[41] Salahuddin H., Nawaz A., Maqsoom A., Mehmood T., Zeeshan B. (2019). Effects of elevated temperature on performance of recycled coarse aggregate concrete. Constr. Build. Mater..

[42] Zega C.J., Di Maio A.A. (2006). Recycled concrete exposed to high temperatures. Mag. Concr. Res..

[43] Cree D., Green M., Noumowé A. (2013). Residual strength of concrete containing recycled materials after exposure to fire: A review. Constr. Build. Mater..

[44] Eguchi K., Teranishi K., Nakagome A., Kishimoto H., Shinozaki K., Narikawa M. (2007). Application of recycled coarse aggregate by mixture to concrete construction. Constr. Build. Mater..

[45] European Committee for Standarisation (2011). Eurocode 2: Design of Concrete Structures-Part 1-2: General Rules-Structural Fire Design.

[46] Chen Z., Chen J., Ning F., Li Y. (2019). Residual properties of recycled concrete after exposure to high temperatures. Mag. Concr. Res..

[47] Varona F.B., Baeza F.J., Bru D., Ivorra S. (2020). Non-linear multivariable model for predicting the steel to concrete bond after high temperature exposure. Constr. Build. Mater..

